# Studies on phytochemical, antioxidant, anti-inflammatory and analgesic activities of *Euphorbia dracunculoides*

**DOI:** 10.1186/s12906-015-0868-0

**Published:** 2015-10-07

**Authors:** Muhammad Majid, Muhammad Rashid Khan, Naseer Ali Shah, Ihsan Ul Haq, Muhammad Asad Farooq, Shafi Ullah, Anam Sharif, Zartash Zahra, Tahira Younis, Moniba Sajid

**Affiliations:** Department of Biochemistry, Faculty of Biological Sciences, Quaid-i-Azam University, Islamabad, Pakistan; Department of Biosciences, COMSATS Institute of Information Technology, Islamabad, Pakistan; Department of Pharmacy, Faculty of Biological Sciences, Quaid-i-Azam University, Islamabad, Pakistan

**Keywords:** Euphorbia dracunculoides, Antioxidant, Anti-inflammatory, Analgesic, Terpenoids, Steroids

## Abstract

**Background:**

Plants provide an alternative source to manage various human disorders due to diverse metabolites. *Euphorbia dracunculoides* of family Euphorbiaceae is used by local practitioners in rheumatism, epilepsy, edema, snake bite, warts and also possesses diuretic and purgative effects. The present study evaluated the antioxidant, anti-inflammatory and analgesic activities of various extracts of *E. dracunculoides*. Further, phytochemical constituents of the leading extracts were also investigated.

**Methods:**

Dry powder of *E. dracunculoides* was extracted with n-hexane (EDH), acetone (EDA), ethanol (EDE), ethanol + water (1:1) (EDEW) and methanol (EDM) and screened for phytochemical classes, total phenolic (TPC) and flavonoid content (TFC). Antioxidant effects of the extracts were manifested by *in vitro* multidimensional assays. The anti-inflammatory and analgesic activities of the extracts were evaluated through carrageenan induced paw edema and hot plate test in rat. In addition, GC-MS analysis of EDH and HPLC-DAD analysis of EDEW was carried out to determine the presence of active constituents.

**Results:**

Qualitative analysis of various extracts of *E. dracunculoides* assured the existence of tannins and coumarins while presence of anthraquinones and anthocyanins was not traced in these extracts. Maximum quantity of TPC and TFC was recorded in EDEW followed by EDE. EDEW and EDE showed significant antioxidant activities with therapeutic potential against hydroxyl and phosphomolybdate radicals, β-carotene bleaching assay and in reducing of iron while moderate to low scavenging abilities were recorded for DPPH, nitric oxide and for iron chelation. During anti-inflammatory activity after 4 h of drug administration the 300 mg/kg body weight dose of EDH (68.660 ± 10.502 %) and EDE (51.384 ± 8.623 %) exhibited strong anti-inflammatory activity and reduced the carrageenan-induced paw edema in rat as compared to standard drug diclofenac sodium (78.823 ± 6.395 %). Treatment of rats with EDH (70.206 ± 5.445 %) and EDE (56.508 ± 6.363 %) after 90 min showed significant increase in percent latency time in hot plate test as compared to morphine (63.632 ± 5.449 %) treatment in rat. GC-MS analysis of EDH indicated the presence of 30 compounds predominantly of steroids and terpenoids. HPLC-DAD analysis against known standards established the presence of rutin, catechin, caffeic acid and myricetin in EDEW.

**Conclusion:**

Our results suggest that presence of various polyphenolics, terpenoids and steroids render *E. dracunculoides* with therapeutic potential for oxidative stress and inflammation related disorders.

## Background

Medicinal plants play an appealing role in modulation of several human disorders. Many civilizations and cultures like Chinese, Ayurvedic, Unani and Hindi has a confirm belief on these cures to treat their health related issues. Plants derived chemicals are important source of clinical agents that have sedative, anti-depressant, antioxidant, antispasmodic, anxiolytic, anti-inflammatory, immunomodulatory, analgesic, anti-pyretic, and cardio protective activities [1]. This customary herbal medicinal system is deep rooted in their cultures and habitats and knowledge of home remedies is conveyed accordingly to their descendants as the time goes [2].

During the normal metabolic process human body produce reactive oxygen species (ROS) and reactive nitrogen species (RNS) as byproduct to accomplish the normal physiological processes. Oxidative stress can be declared as a root cause for many diseases especially chronic inflammatory disorders such as rheumatism, diabetes, carcinomas, mutagenesis, sarcomas, aging and circulatory disorders [3]. Living cells possess an excellent defense mechanism to cope with damaging effects of free radicals and this is made possible by a variety of detoxifying enzymes and metabolites inside the body that scavenge free radicals in a professional way. Among them superoxide dismutase (SOD), catalase (CAT) and peroxidase (POD) are the main stream enzymes supported with scavengers and chelating agents. The indirectly acting antioxidants are very important in protecting body from deleterious effects of ROS/RNS. This group comprised thousands of compounds involved in free radical scavenging and most of them are derived from dietary sources such as vegetables and fruits.

We are much more endangered by free radicals than being secured by natural scavenging entities producing in our body in stressed conditions. Phytochemicals play a unique role in avoiding hazardous effects of free radicals [4]. Polyphenols are excellent free radical scavengers and inhibitors of lipid peroxidation. Terpenoids and steroids are useful metabolites for various metabolic disorders. These properties make their role crucial from therapeutic and pharmacologic point of views.

The trend of finding the pharmacological activities of the plants of known medicinal uses in folk medicines is quite much effective than going randomly after it [5, 6]. The genus *Euphorbia* contributes as the largest amongst the spurge family with over 2000 species, with awesome use value in folk Chinese medicinal system used mainly for skin diseases and edemas [7].

Several species of *Euphorbia* have been used in local system of medicine; for the treatment of various ailments. Rhizome of *Euphorbia neriifolia* and aerial parts of *Euphorbia royleana* has been used for the treatment of anti-inflammatory disorders [8, 9]. In local system of medicine such as Africa and Australia, *Euphorbia hirta* has used as a remedy for various ailments especially in hypertension and edema. Previous studies have evaluated *E. hirta* for antipyretic, analgesic, anti-inflammatory and diuretic activities [10, 11]. Strong antioxidant activity of *E. macroclada* and *E. acanthothamnos* has been determined in previous studies [12].

*Euphorbia dracunculoides* Lam., (Euphorbiaceae) is distributed in Southwest Asia, North Africa and South Europe. It is an annual herb and is usually found along riverbanks, in valleys and roadsides of sandy areas in Khyber Pakhtunkhwa Province of Pakistan. Fruits are used to remove warts from skin [13]. Leaves are used in snake bite and epilepsy [14]. A decoction of whole plant is applied on body of cattle for lice killing [15]. *Euphorbia dracunculoides* has been used by local practitioners for its diuretic and purgative properties. Structurally diversified 19 diterpenoids have been isolated from aerial parts of *E. dracunculoides* [16]. Because of similar morphology of the dried aerial parts of *E. drancunculoides* to *Ruta graveolens*, it is sold or used clinically as replacement of *R. graveolens* for analgesic and inflammatory disorders; gout and arthritis [17, 18]. To our knowledge the scientific validation of *E. dracunculoides* for the use in inflammation related disorders has not been reported earlier. For this purpose we investigated preliminary phytochemical composition, antioxidant and anti-inflammatory activities of various extracts of *E. dracunculoides*. EDEW showed significant antioxidant activity; was subjected to HPLC-DAD analysis for the presence of flavonoid constituents. EDH exhibited remarkable analgesic and anti-inflammatory activities; was explored for GC-MS analysis.

## Methods

### Preparation of extract

Plant was recognized by its local name and collected from the District Lakki Marwat in March 2014. The plant sample after authentication was deposited (Accession No. 127962) at the Herbarium of Pakistan, Quaid-i-Azam University, Islamabad, Pakistan. The fully shade dried aerial parts of *E. dracunculoides* were first powdered followed by two extraction (36 h) with n-hexane, acetone, ethanol, ethanol + water (1:1 v/v) and 95 % methanol in 2 : 1 ratio (v/w). Filtered extracts; EDH, EDA, EDE, EDEW and EDM were dried under vacuum in a rotary evaporator at 40 °C and stored at 4 °C for *in vitro* and *in vivo* experiments.

### Phytochemical analysis

Different qualitative tests were employed to identify the phytochemical classes present in various extract of the plant *E. dracunculoides*.

### Assessment of phenols

For the presence of phenols previously reported methodology was followed [19]. Each sample (1 mg) was suspended in 2 ml of distilled water containing 10 % ferric chloride. The confirmation sign for the presence of phenol was the development of blue or green color.

### Assessment of flavonoids

Protocol of Trease and Evans [20] was followed to establish the presence of flavonoids in each sample. Briefly, 1 mg of each sample was allowed to react with 1 ml of 2 N sodium hydroxide and appearance of yellow color was considered as the confirmation sign of flavonoid presence.

### Assessment of coumarins

An aliquot of each sample (1 mg/ml) was mixed with 1 ml of 10 % sodium hydroxide. Appearance of yellow color formation in the test tube was the proof of coumarins presence in test sample [19].

### Assessment of saponins

Each sample (2 mg) was suspended in 2 ml of distilled water and vigorously shaken. The formation of a soapy layer of almost 1–2 cm was the indication of saponins presence [19].

### Assessment of tannins

Confirmative sign of tannins was the development of dark blue or greenish black color on the mixing of 1 mg of each sample and 2 ml of 5 % ferric chloride [20].

### Assessment of terpenoids

Each sample 0.5 mg was mixed with 2 ml of chloroform and 2 ml of concentrated sulphuric acid. The appearance of a red brown colored layer in the middle of two layers confirmed the existence of terpenoids [20].

### Assessment of anthraquinone

Development of red color was considered as indication for the presence of anthraquinone after mixing of 1 mg of each sample with 2 ml of diluted 2 % hydrochloric acid [19].

### Assessment of anthocyanin and betacyanin

Each sample (1 mg) was boiled for 10 min in 2 ml of 1 N sodium hydroxide. Formation of bluish green color was the sign of anthocyanin and yellow color formation of betacyanin presence [20].

### Assessment of alkaloids

An amount of 2 mg of each sample was mixed with concentrated sulphuric acid. The reaction mixture was allowed to react with Mayer’s reagent. Appearance of green color or formation of white precipitates was the symbol of alkaloid presence [20].

### Quantitative analysis

Total phenolic as well as flavonoid contents were quantified by the following narrated procedures.

### Total phenolic contents (TPC)

To determine the total phenolic content in each sample spectrophotometric method already reported was followed [21]. Briefly, 1 ml of each sample (1 mg/ml) was mixed with 9 ml of distilled water and I ml of Folin-Ciocalteu reagent. The mixture obtained was mixed rigorously for 5 min and 10 ml of 7 % Na_2_CO_3_ was added to the mixture. By the addition of distilled water the final volume of mixture was made to 25 ml and was incubated at room temperature for 90 min. Optical density was ensured at wavelength of 750 nm in triplicate for each sample. By using gallic acid as standard the estimation of TPC was carried out as mg of gallic acid equivalents (GAE) per gram of dry extract/fraction.

### Total flavonoid content (TFC)

To estimate the TFC in the test samples, 0.3 ml of each sample was mixed with 0.15 ml of 0.5 M NaNO_2_ followed by the addition of 0.1 ml of 0.3 M AlCl_3_.6H_2_O, and 3.4 ml of 30 % methanol [22]. An aliquot of 1 ml of 1 M NaOH was added to it after a lapse of 5 min. The optical density of the reaction mixture was recorded at 506 nm wavelength against the reagent blank. Total flavonoid content as mg rutin equivalents per gram of dry extract/fraction was estimated while using the calibration curve of rutin.

### Gas chromatography-Mass spectrometry (GC-MS) analysis

EDH was analyzed for the presence of active constituents on “Thermo GC-Trace Ultra Ver; 5.0” gas chromatograph coupled with a “Thermo MS DSQ II” for mass determination. Components were separated on a “ZB 5-MS Capillary Standard Non-polar Column” with 60 m length having 0.25 μm film thickness. During the experiment the temperature was raised from 70 to 260 °C at a rate of 6 °C/min. The flow rate of the carrier gas; helium was 1 ml/min while injection volume of the samples was 1 μl. The identification of chemical constituents was based on comparison of their relative retention times and mass spectra with those obtained from authentic sample and/or the NIST/NBS and Wiley libraries spectra [23].

### High performance liquid chromatography (HPLC) analysis

On account of significant antioxidant activity for most of the *in vitro* assays the EDEW extract was selected for HPLC-DAD analysis. HPLC analysis of EDEW was carried out by using HPLC-DAD (Agilent Germany) equipment using Sorbex RXC8 (Agilent USA) analytical column with 5 μm particle size and 25 ml capacity. Mobile phase was consisted of eluent A, (acetonitrile-methanol-water-acetic acid /5: 10: 85: 1) and eluent B (acetonitrile-methanol-acetic acid/40: 60: 1). The gradient (A: B) utilized was the following: 0–20 min (0 to 50 % B), 20–25 min (50 to 100 % B), and then isocratic 100 % B (25–40 min) at flow rate of 1 ml/min. The injection volume of the sample was 20 μl. Before the injection samples were filtered through 0.45 μm membrane filter. Among the standards rutin and gallic acid were analyzed at 257 nm, catechin at 279 nm, caffeic acid at 325 nm and quercetin, myricetin, kampferol were analyzed at 368 nm [24]. Each time the column was reconditioned for 10 min before the next analysis. All samples were assayed in triplicates. Quantification was carried out by the integration of the peak using the external standard method. All chromatographic operations were carried out at an ambient temperature.

### *In vitro* antioxidant assays

The *in vitro* antioxidant assays were carried out by preparing the plant samples (1 mg/ml) in 95 % methanol and then making its serial dilutions. The specific protocol was followed for finding specific scavenging activities of the plant samples.

#### DPPH (1, 1-diphenyl-2-picryl-hydrazyl) radical scavenging assay

The DPPH scavenging capabilities of deleterious effects of free radical were determined by following the methodology of [25]. An amount of 24 mg of DPPH was dissolved in 100 ml methanol and the stock solution was kept at 20 °C temperature for further use. The optical density of DPPH was optimized at 0.908 (±0.02) at 517 nm by diluting the pre made DPPH stock solution with methanol. An aliquot of 3 ml of DPPH was mixed with 100 ml plant samples with different concentrations (25–250 μg/ml). Following continuous stirring the test tubes were incubated for 15 min at room temperature. Optical density was recorded at wavelength of 517 nm. Ascorbic acid was used as standard to compare the antioxidant activity. Antioxidant capacity was determined by the following equation:

$$ \mathrm{Inhibition}\ \% = \left[\frac{\mathrm{Absorbance}\ \mathrm{of}\ \mathrm{control}-\mathrm{Absorbance}\ \mathrm{of}\ \mathrm{the}\kern0.5em \mathrm{sample}}{\mathrm{Absorbance}\ \mathrm{of}\ \mathrm{control}}\right]\times 100 $$

#### Hydroxyl radical scavenging assay

The power of scavenging hydroxyl free radicals refers to the antioxidant potential of plant samples using methodology practiced by [26]. This technique involved the mixing of 500 μl of 2-deoxyribose (2.8 mM) prepared in 50 mM phosphate buffer and its pH was maintained at 7.4. The reaction mixture was prepared by addition of 100 μl of 0.1 M EDTA, 200 μl of ferric chloride (100 mM) and 100 μl of 200 mM H_2_O_2_ and 100 μl of plant sample.

The initiation of reaction was brought by the introduction of 100 μl of ascorbic acid (300 mM) and incubated for 1 h at 37 °C. Then 1 ml of 2.8 % trichloroacetic acid and 1 ml of 1 % w/v thiobarbituric acid prepared in 50 mM NaOH were added to the reaction mixture. The whole recipe was heated in water bath for 15 min. After cooling to room temperature the absorbance of the reaction mixture was recorded at 532 nm. The hydroxyl radical scavenging activity was analyzed by the following formula:

$$ \mathrm{Scavenging}\ \mathrm{effect}\ \% = \left[\frac{1-\mathrm{Absorbance}\ \mathrm{of}\ \mathrm{the}\kern0.5em \mathrm{sample}}{\mathrm{Absorbance}\ \mathrm{of}\ \mathrm{control}}\right]\times 100 $$

#### Nitric oxide scavenging assay

The method of Bhaskar and Balakrishnan [27] was used to assess the nitric oxide scavenging potential of the plant samples. The Griess reagent was prepared by adding equimolar quantity of 0.1 % napthylenediamine in distilled water and 1 % of sulphanilamide in 5 % phosphoric acid. A volume of 100 μl of sample was added to 100 μl of sodium nitroprusside (10 mM) being prepared in saline phosphate buffer. An aliquot of 1 ml of the Griess reagent was added to the reaction mixture. After incubation at room temperature for 3 h the optical density of the reaction mixture was noted spectrophotometrically at 546 nm using ascorbate as a positive control. Following formula was used for determining the percentage inhibition of nitric oxide radical formation.

$$ \mathrm{Inhibition}\ \% = \left[\frac{\mathrm{Absorbance}\ \mathrm{of}\ \mathrm{control}-\mathrm{Absorbance}\ \mathrm{of}\ \mathrm{the}\kern0.5em \mathrm{sample}}{\mathrm{Absorbance}\ \mathrm{of}\ \mathrm{control}}\right]\times 100 $$

#### Chelating power assay

The iron (II) binding capability at multiple sites confers the antioxidant potential of plant samples [28]. The plant sample with its serial dilutions was prepared in methanol and 200 μl of each dilution was mixed with 900 μl of methanol and 100 μl FeCl_2_.2H_2_O (2.0 mM) and incubated for 5 min. The reaction was initiated by introducing 400 μl of ferrozine (5.0 mM). After incubation for 10 min the optical density was recorded at 562 nm using EDTA as standard in comparison. The chelating power was determined by the following formula:

$$ \mathrm{Chelating}\ \mathrm{effect}\ \% = \left[\frac{\mathrm{Absorbance}\ \mathrm{of}\ \mathrm{control}-\mathrm{Absorbance}\ \mathrm{of}\ \mathrm{the}\kern0.5em \mathrm{sample}}{\mathrm{Absorbance}\ \mathrm{of}\ \mathrm{control}}\right]\times 100 $$

#### β-Carotene bleaching assay

Inhibition ability of β-carotene bleaching was recorded by the method of [29]. The reagent was prepared by dissolving 2 mg of β-carotene in 10 ml of chloroform followed by addition of 200 mg of Tween 80 and 20 mg of linoleic acid. After evaporation of chloroform, 50 ml of distilled water was added to the reaction mixture and vigorously vortexed to get a uniform emulsion of β-carotene linoleate. In freshly prepared 250 μl of emulsion, 30 μl of plant sample was added and optical density was measured at 470 nm at 0 h. Then after keeping the reaction mixture at 45 °C for 2 h the final optical density was again recorded. Catechin was served as standard in this assay and % inhibition of β-carotene was determined by the formula:

$$ \%\ \mathrm{inhibition} = \left[\left({\mathrm{A}}_{\mathrm{A}(120)}\hbox{--} {\mathrm{A}}_{\mathrm{C}\ (120)}\right)/\left({\mathrm{A}}_{\mathrm{C}\ (0)}\hbox{--} {\mathrm{A}}_{\mathrm{A}(120)}\right)\right] \times 100 $$

Where A_A_ (120) is the absorbance of the antioxidant at t = 120 min, A_C_ (120) is the absorbance of the control at t = 120 min, and A_C_ (0) is the absorbance of the control at t = 0 min.

#### Reducing power assay

Reducing power of various extracts was estimated by the method of [30, 31]. Briefly, 2 ml of plant extract was mixed with 2 ml of 0.2 M phosphate buffer (pH 6.6) and 2 ml of potassium ferricyanide (10 mg/l) and the reaction mixture was incubated at 50 °C for 20 min. After addition of 2 ml of trichloroacetic acid (100 mg/l) in the reaction mixture, 2 ml was of it was diluted with 2 ml of distilled water and 0.4 ml of FeCl_3_ (0.1 %). Optical density of the reaction mixture was measured at 700 nm after 10 min of incubation. Gallic acid was used as standard.

#### Phosphomolybedenum assay

The methodology of [31] was used to assess the antioxidant capabilities of the plant sample. Accordingly, 0.1 ml of the plant sample was mixed with 1 ml of the reagent solution (prepared by adding 28 mM Na_3_PO_4_ and 0.6 M H_2_SO_4_ with that of 4 mM ammonium molybdate). After incubation at 95 °C in a water bath for 90 min the reaction mixture was cooled down to room temperature and optical density was recorded at 765 nm. Ascorbic acid served as standard in this assay.

### Anti-inflammatory activity

Protocol of [32] was followed to estimate the anti-inflammatory activity of extracts by carrageenan-induced paw edema in rat. Sprague-Dawley rats (six weeks old) weighing about 150–200 g were randomly divided into six groups containing 6 rats in each group. Rats had free access to the laboratory feed and water. National institute of health (NIH) guidelines were strictly carried out using test animals for experimentation. Ethical Committee of Quaid-i-Azam University Islamabad approved the study protocol (Bch#0267) for the animal care and experimentation. Group I was negative control and received DMSO, Group II orally received 10 mg/kg of drug diclofenac sodium (1:1 W/V DMSO). Group III to Group VII were given 300 mg/kg dose of EDH, EDA, EDE, EDEW and EDM respectively. To develop the paw edema, 1 ml/kg body weight of carrageenan solution (0.9 % w/v; saline) was injected in the right paw of each rat after 1 h of the administration of plant sample. Paw volume was measured plethysmographically at 0, 1st, 2nd, 3rd and 4th h after carrageenan injection and the following formulae for calculating percent inhibition of edema were used;

$$ \mathrm{E}\mathrm{V}=\mathrm{P}\mathrm{V}\mathrm{A}-\mathrm{P}\mathrm{V}\mathrm{I} $$

Where, EV = Edema volume, PVI = Paw volume before carrageenan administration (i.e. initial paw volume) and, PVA = Paw volume after carrageenan administration.

$$ \mathrm{Percent}\ \mathrm{inhibition}=\left[\frac{\left(\mathrm{E}\mathrm{V}\mathrm{c}\ \hbox{--}\ \mathrm{E}\mathrm{V}\mathrm{t}\right)\ }{\left(\mathrm{E}\mathrm{V}\mathrm{c}\right)} \times 100\right]. $$

EVc = Edema volume of control animals, EVt = Edema volume of test sample animals.

### Analgesic activity

The procedure described by [33], was followed to perform this test. Sprague-Dawley rats of either sex (*n* = 6) weighing 180–220 g were used. Animals were subjected to pre-testing on a hot plate analgesimeter (Harvard apparatus Ltd., UK) maintained at 55 ± 0.1 ^o^C. Animals having latency time greater than 15 s on hot plate during pre-testing were excluded. Animals were divided randomly into 7 groups, each consisting of six rats. Group I was negative control and received 2 ml/kg p.o. DMSO, Group II received 10 mg/kg i.p. of the standard drug morphine sulphate. Group III to Group VII were given 300 mg/kg p.o. dose of EDH, EDA, EDE, EDEW and EDM respectively. The latency time was recorded for each group at 0, 30, 60 and 90 min following drug administration. In order to prevent the tissue damage the cut off time of 30 s was set for all animals. Percent analgesia was calculated using the following formula.

$$ \%\ \mathrm{Analgesia}=\left[\frac{\left(\mathrm{Test}\ \mathrm{latency}\ \hbox{--}\ \mathrm{control}\ \mathrm{latency}\right)\ }{\left(\mathrm{Cut}\ \mathrm{off}\ \mathrm{time}-\mathrm{control}\ \mathrm{latency}\right)} \times 100\right] $$

### Statistical analysis

Data obtained in this study was presented as mean ± SD. One way analysis of variance was performed to determine the variability among groups by Statistix 8.1. GraphPad Prim 5 was used to determine the correlation of IC_50_ values of antioxidant assays with TPC and TFC by Pearson’s correlation coefficient. Significant differences among groups were calculated by Tukey’s multiple comparison tests. Statistical significance was set at *P* > 0.05.

## Results

### Extraction yield, total phenolic and flavonoid content

By using 100 g of dry powder of *E. dracunculoides* for extraction, the maximum yield 5462 mg powder was obtained for EDEW followed by 1703 mg (EDM), 1340 mg (EDA), 724 mg (EDE) and 592 mg (EDH). On the basis of standard regression lines for gallic acid (y = 0.0103x + 0.1875; R^2^ = 0.9978) and rutin (y = 0.00028x + 0.497; R^2^ = 0.998), the equivalents of TPC and TFC were calculated (Table 1). EDEW showed maximum quantity of TPC (17.35 ± 0.62 mg GAE/g dry sample) followed by EDE (16.41 ± 0.54 mg GAE/g dry sample), EDM (14.11 ± 0.37 mg GAE/g dry sample), EDA (10.62 ± 0.33 mg GAE/g dry sample) and EDH (8.21 ± 0.49 mg GAE/g dry sample). Flavonoids were found to be rich in EDEW (7.57 ± 0.42 mg RE/g dry sample) followed by EDE (6.77 ± 0.31 mg RE/g dry sample), EDM (4.84 ± 0.29 mg RE/g dry sample), EDA (4.52 ± 0.37 mg RE/g dry sample) and EDA (4.18 ± 0.25 mg RE/g dry sample).

**Table 1:** Extraction yield, total phenolic and flavonoid contents of *E. dracunculoides*

Extract	Yield (mg/100 g powder)	Total phenolic content (mg gallic acid equivalent/ g dry sample)	Total flavonoid content (mg rutin equivalent/ g dry sample)
EDH	592	8.21 ± 0.49^e^	4.18 ± 0.25^e^
EDA	1,340	10.62 ± 0.33^d^	4.52 ± 0.37^d^
EDE	724	16.41 ± 0.54^b^	6.77 ± 0.31^b^
EDEW	5,462	17.35 ± 0.62^a^	7.57 ± 0.42^a^
EDM	1,703	14.11 ± 0.37^c^	4.84 ± 0.29^c^

*EDH E. dracunculoides* n-hexane extract, *EDA E. dracunculoides* aqueous extract, *EDE E. dracunculoides* ethyl acetate, *EDEW E. dracunculoides* ethyl acetate + water extract, *EDM E. dracunculoides* methanol extract. Each value is represented as mean ± SD (*n* = 3). Means with different superscript (^a-e^) letters in the column are significantly (*P* < 0.05) different from one another

### Phytochemical classes

The results of phytochemical analysis of all the extracts of *E. dracunculoides* are listed in Table 2. Qualitative analysis of *E. dracunculoides* ensured the presence of tannins, phenols, flavonoids and coumarins in all extracts of *E. dracunculoides* except coumarins were absent in EDH. Presence of anthraquinones was not recorded in all the extracts. EDEW contained the maximum phytochemical classes and EDA showed the least number of existent phytochemical classes.

**Table 2:** Phytochemical analysis of *E. dracunculoides*

Phytochemical	EDH	EDA	EDE	EDEW	EDM
Terpenoids	++	-	-	+	-
Coumarins	-	+	++	+++	+++
Flavonoids	+	+	+++	+++	++
Tannins	+	++	++	+++	+++
Anthraquinones	-	-	-	-	-
Phenols	+	+	+++	+++	++
Alkaloids	+	-	-	++	++
Saponins	-	-	-	++	-
Betacyanin	-	-	+	++	++

(+) present, (-) absent, (++) moderate concentration, (+++) abundant concentration

*EDH E. dracunculoides* n-hexane extract, *EDA E. dracunculoides* aqueous extract, *EDE E. dracunculoides* ethyl acetate, *EDEW E. dracunculoides* ethyl acetate + water extract, *EDM E. dracunculoides* methanol extract

### GC-MS analysis of n-hexane extract

**Fig. 1:**
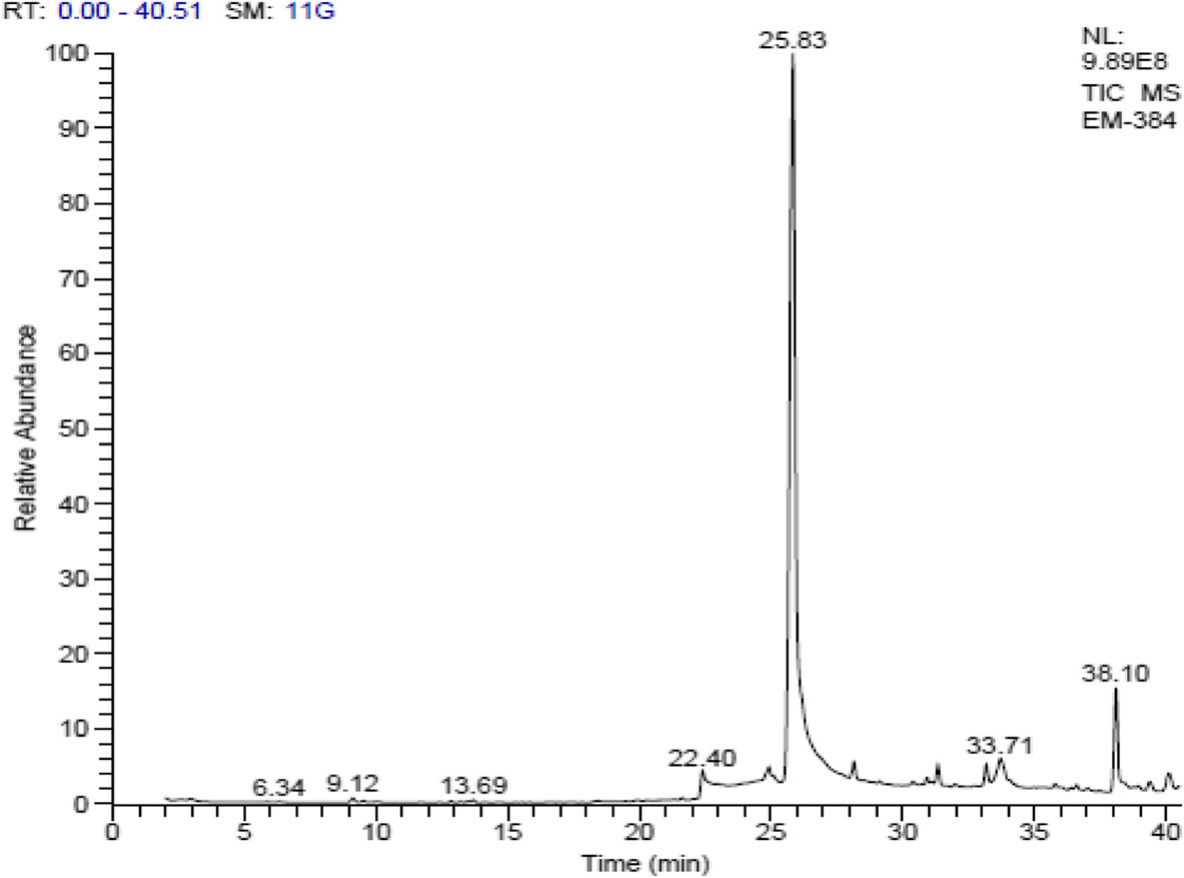
GC-MS analysis of n-hexane extract of *E. dracunculoides*

The *n-*hexane extract of *E. dracunculoides* was selected for GC-MS analysis due to its significant analgesic and anti-inflammatory activities. GC-MS analysis indicated that EDH contained 30 chemical constituents eluted between 6.36 and 40.10 min (Fig. 1). The identification of chemical constituents was based on comparison of their relative retention times and mass spectra with those obtained from authentic sample and/or the NIST/NBS and Wiley libraries spectra (Table 3). Of these 30 chemical constituents, there were 7 terpenoides (13.25 % on the basis of peak area), 4 lactones (2.12 %), 3 steroids (2.59 %), 3 phenols (1.60 %), 2 hetrocycles (73.91 %), 1 fatty acid (2.63 %), 1 ester (1.30 %), 1 carboxylic acid (0.58 %), 1 lactam (0.51 %), 1 nitrile (0.40 %), 1 alkyne (0.36 %), 1 hydrazone (0.19 %), 1 terpene (0.18 %), 1 aldehyde (0.14 %), 1 ketone (0.13 %) and 1 amino acid (0.1 %) in EDH.

**Table 3:** GC-MS analysis of EDH

S. No	Compound name	Type
1	O,N-Permethylated N-Acetyllysine	Amino acid
2	Phenol, 2-Methyl-5-(1-Methylethyl)-	Phenol
3	2,4-Decadienal, (E,E)- (Cas)	Aldehyde
4	Phenol, 2-Methoxy-4-(2-Propenyl)- (Cas)	Phenol
5	3,4-Dihydro-2H-1,5-(3″-t-butyl) benzodioxepine	Heterocycle
6	4-(Phenylethynyl)-1-methoxybenzene	Alkyne
7	(-)-Loliolide	Lactone
8	2-Pentadecanone, 6,10,14-trimethyl-	Terpenoid
9	(E,E)-Farnesyl Acetone	Terpenoid
10	Hexadecanoic acid, methyl ester (CAS)	Terpenoid
11	Hexadecanoic acid (CAS)	Fatty acid
12	Hexadecanoic acid, ethyl ester	Terpenoid
13	9,12,15-Octadecatrienoic acid, methyl ester, (Z,Z,Z)-(CAS)	Terpenoid
14	1H-Imidazole, 1-ethyl- (CAS)	Heterocycle
15	3-(2-Methylaminophenyl)-1H-benzopyran-1-one	Lactone
16	4,8,12,16-Tetramethylheptadecan-4-olide	Lactone
17	Estra-1,3,5(10)-trien-17-one, 3-hydroxy-2-methoxy-	Steroid
18	3à,6á-Dihydroxyandrost-4-Ene-17-One	Steroid
19	Di-(2-ethylhexyl)phthalate	Ester
20	10-Methylundeca-2,4,8-triene	Terpene
21	Austrobailignan-6	Phenol
22	Butyl 9,12,15-octadecatrienoate	Terpenoid
23	1,7,7-trimethyl-3-thiocyanatomethylen-bicyclo[2,2,1]hepta	Ketone
24	2-Acetamido-3-(3,4-dihydroxyphenyl) propenoic acid	Carboxylic acid
25	2,7-Diphenyl-1,6-dioxopyridazino[4,5-2′,3′] pyrrolo[4′,5′-D pyridazine	Lactam
26	4-(cis-6-Methoxymethyl-3,4-dimethyl-3-cyclohexenyl)-trans-3-buten-2-one 2,4-dinitrophenylhydrazone	Hydrazone
27	1-Heneicosyl formate	Terpenoid
28	2-Methoxy-6-hydroxy-1,3-dicyanoazulene	Nitrile
29	Xanthinin	Lactone
30	Stigmasta-5,22-dien-3-ol, (3á,22E)- (CAS)	Steroid

### HPLC-DAD analysis of ethanol + water extract

**Fig. 2:**
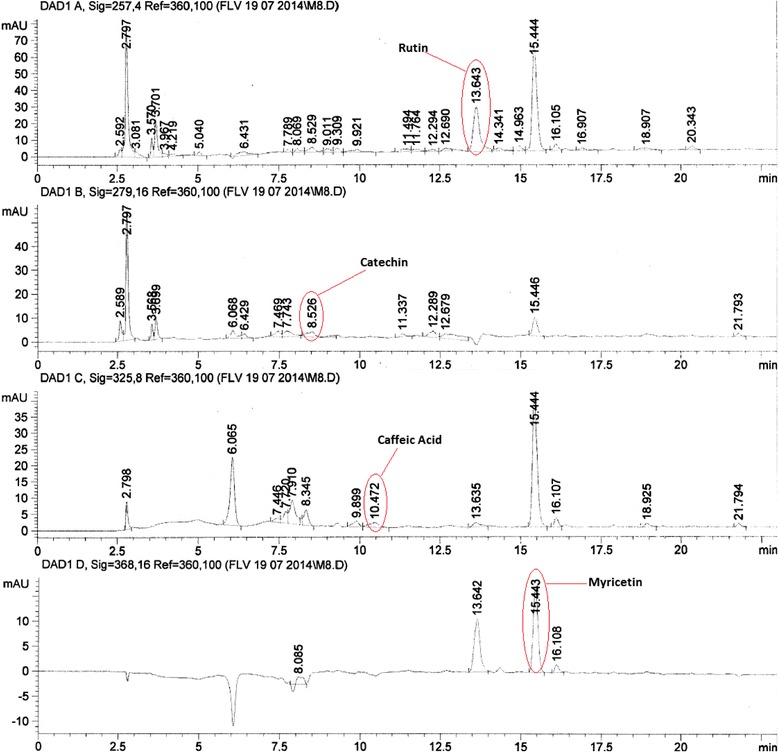
HPLC-DAD profile of *E. dracunculoides* ethanol + water extract (EDEW) at different wavelengths. Signal 1: 257λ, Signal 2:279λ, Signal 3: 325λ, Signal 4; 368λ.Conditions: Mobile Phase A-ACN: MEOH: H2O: AA:: 5:10:85:1, Mobile phase B-ACN: MEOH: AA:: 40:60:1, Injection volume 20 μL, Flow rate 1 ml/min, Agilent RP-C8

HPLC-DAD profile of EDEW was illustrated in Fig. 2. EDEW showed the existence of rutin, catechin, caffeic acid and myricetin. Rutin showed maximum quantity (65.8 ± 2.2 μg/mg dry extract) followed by catechin (15.3 ± 1.2 μg/mg dry extract), caffeic acid (8.5 ± 0.75 μg/mg dry extract) and myricetin (4.54 ± 0.35 μg/mg dry extract) as demonstrated in Table 4.

**Table 4:** HPLC-DAD results for EDEW of *E. dracunculoides*

Flavonoid/Phenolics	Signal wavelength	Quantity (μg/mg dry extract)
Rutin	257	65.8 ± 2.2
Catechin	279	15.3 ± 1.2
Caffeic acid	325	8.5 ± 0.75
Myricetin	368	4.54 ± 0.35

*EDEW* ethanol + water extract. Each value is represented as mean ± SD (*n* = 3)

### *In vitro* antioxidant activities

#### DPPH radical scavenging activity

Moderate to low scavenging activity for DPPH was exhibited by all the extracts of *E. dracunculoides* (Table 5). Minimum IC_50_ values were exhibited by EDEW (144.7 ± 3.4 μg/ml) followed by EDE (263.6 ± 3.7 μg/ml) and EDM (351.3 ± 4.5 μg/ml) while EDA and EDH showed the higher IC_50_ values (> 1000 μg/ml). Overall, order of IC_50_ of EDEW < EDE < EDM < EDA < EDH was observed. The DPPH radical scavenging activity of extract/fractions showed significant correlation with TPC (R^2^ = 0.9028, *P* < 0.01) and non-significant with TFC (R^2^ = 0.5687, *P* > 0.05, Table 6). All the extracts showed the higher IC_50_ values than ascorbic acid (26.65 ± 2.4 μg/ml). However, concentration dependent activity was recorded for all the extracts.

**Table 5:** IC_50_ values of different antioxidant activities of *E. dracunculoides* extracts

IC_50_(μg/ml)					
Plant sample	DPPH scavenging	Hydroxyl scavenging	Nitric Oxide	β-Carotene	Iron chelating
EDH	>1000	297.12 ± 3.22^a^	756.31 ± 4.23^a^	244.06 ± 3.81^a^	616.57 ± 3.33^a^
EDA	>1000	202.20 ± 3.41^b^	583.40 ± 3.65^b^	181.10 ± 1.93^c^	531.43 ± 2.94^b^
EDE	263.61 ± 3.71^b^	121.70 ± 2.05^c^	366.81 ± 3.12^d^	120.53 ± 2.52^d^	279.51 ± 2.57^e^
EDEW	144.71 ± 3.44^c^	78.02 ± 1.21^d^	317.80 ± 3.35^e^	100.40 ± 1.81^e^	331.46 ± 2.42^d^
EDM	351.33 ± 4.54^a^	142.43 ± 2.51^c^	405.01 ± 3.57^c^	210.23 ± 3.41^b^	446.3 ± 3.52^c^
Rutin	-	80.74 ± 2.25^d^	-	-	-
AA	26.65 ± 2.41^d^	-	244.45 ± 2.35^f^	-	-
Gallic acid	-	45.97 ± 1.92^e^	-	-	-
EDTA	-	-	-	-	189.85 ± 1.53^f^
Catechin	-	-	-	78.57 ± 2.15^f^	-

*EDH E. dracunculoides* n-hexane extract, *EDA E. dracunculoides* aqueous extract, *EDE E. dracunculoides* ethyl acetate, *EDEW E. dracunculoides* ethyl acetate + water extract, *EDM E. dracunculoides* methanol extract

Values are presented as means ± SD (*n* = 3). Means with different superscript (^a-f^) letters in the column are significantly (*P* < 0.01) different from one another

**Table 6:** Correlation of IC_50_ values of different antioxidant activities of *E. dracunculoides* with total phenolic and total flavonoid contents

Antioxidant Activity	Correlation R^2^	
	TFC	TPC
DPPH radical scavenging activity	0.9028^**^	0.5687
Hydroxyl radical scavenging activity	0.9505^**^	0.7224^*^
Iron chelating assay	0.9361^**^	0.8232^*^
Nitric Oxide radical scavenging Activity	0.9638^**^	0.6822^*^
β - carotene bleaching scavenging activity	0.7579^*^	0.8965^**^
Phosphomolybdenum assay	0.7429^*^	0.893^**^
Reducing power assay	0.9812^**^	0.7349^*^

Column with different superscripts are significantly different ^*, **,^ indicate *P <* 0.05, *P* < 0.01. *TFC* total flavonoid content, *TPC* total phenolic content

#### Hydroxyl radical (•OH) scavenging assay

All the extracts of *E. dracunculoides* scavenged •OH radicals and prevented 2-deoxyribose breakdown (Table 5). A concentration-dependent pattern was observed for hydroxyl radical scavenging activity. Lowest IC_50_ values were recorded for EDEW (78.02 ± 1.21 μg/ml) followed by EDE (121.70 ± 2.05 μg/ml), EDM (142.43 ± 2.51 μg/ml), EDA (202.20 ± 3.4 μg/ml) and EDA (297.12 ± 3.22 μg/ml). IC_50_ values of EDH, EDA, EDE, EDEW and EDM were significantly higher from rutin (80.74 ± 2.25 μg/ml) and gallic acid (45.97 ± 1.92 μg/ml). Significant correlation of IC_50_ values of hydroxyl radical scavenging was determined for TPC (R^2^ = 0.9505, *P* < 0.01) as well as for TFC (R^2^ = 0.7224, *P* <0.05) (Table 6).

#### Nitric oxide (NO^−^) scavenging assay

In the present study, moderate level of nitric oxide scavenging activity was observed for all the extracts with IC_50_ values for EDEW (317.8 ± 3.35 μg/ml), EDE (366.81 ± 3.12 μg/ml) and EDM (405.01 ± 3.57 μg/ml) as compared to standard ascorbic acid (244.45 ± 2.35 μg/ml). IC_50_ values for other extracts were 583.4 ± 3.65 μg/ml and 756.31 ± 4.23 μg/ml for EDA and EDH, respectively (Table 5). IC_50_ values obtained for nitric oxide scavenging activity exhibited a significant correlation with TPC (R^2^ = 0.9638, *P* <0.01) and TFC (R^2^ = 0.6822, *P* <0.05) (Table 6).

#### Iron chelating activity

Iron chelating activity (IC_50_ values) of *E. dracunculoides* extracts are given in Table 5. In current study, the best IC_50_ values for iron chelation were exhibited by EDE (279.51 ± 2.57 μg/ml) while the least by EDH (616.57 ± 3.3 μg/ml). IC_50_ value for other extracts was; EDEW (331.46 ± 2.42 μg/ml), EDM (446.3 ± 3.52 μg/ml) and EDA (531.43 ± 2.94 μg/ml). IC_50_ of standard EDTA was 189.85 ± 1.53 μg/ml (Table 5). Significant correlation (R^2^ = 0.9361, *P* < 0.01) of IC_50_ values was observed with TFC (R^2^ = 0.9361, *P* < 0.01) and for TPC (R^2^ = 0.8232, *P* < 0.05) (Table 6 ).

#### β-Carotene scavenging activity

Ethanol + water extract of *E. dracunculoides* (EDEW) showed the lowest IC_50_ value (100.40 ± 1.8 μg/ml) as compared to other extracts viz. EDE (120.53 ± 2.52 μg/ml), EDM (210.23 ± 3.41 μg/ml) and EDA (181.10 ± 1.9 μg/ml). However, the maximum IC_50_ was shown by EDH (244.06 ± 3.8 μg/ml) as compared to the standard catechin (78.57 ± 2.15 μg/ml) shown in Table 5. The concentration dependent inhibition in β-carotene bleaching power pattern was observed for all the extracts. The assay showed significant correlation of IC_50_ with both TPC (R^2^ = 0.7579, *P* < 0.05) and TFC (R^2^ = 0.8965, *P* < 0.01) as shown in Table 6.

#### Reducing power assay

**Fig. 3:**
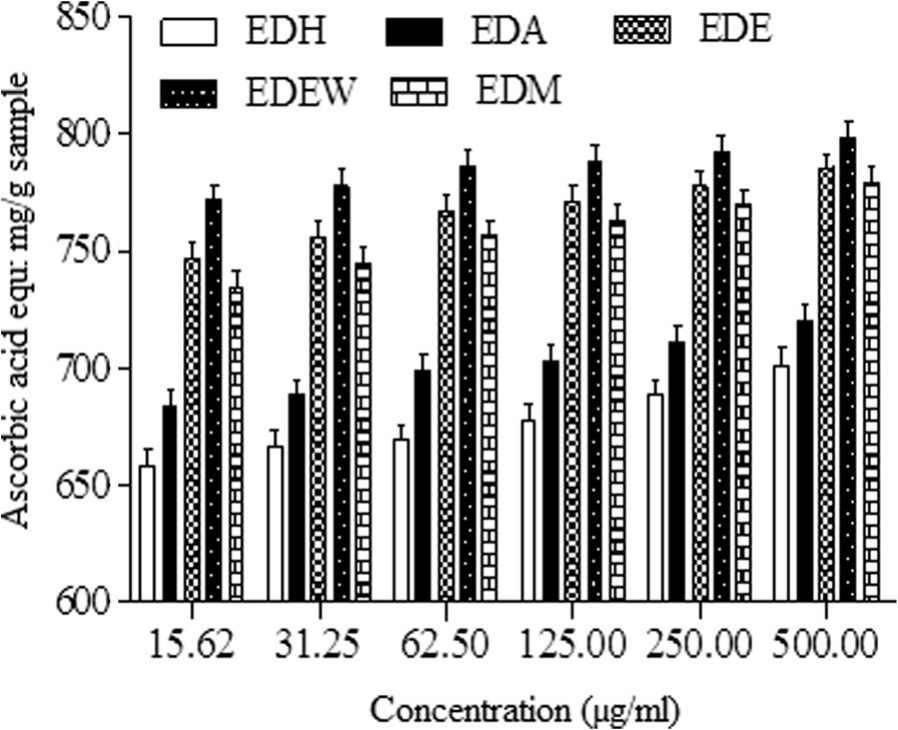
Reducing power activity of different concentrations of *Euphobia dracunculoids* extracts

Ethanol + water extract (EDEW) showed the highest reducing power with 792.59 mg ascorbic acid equivalent/g sample measured at 250 μg/ml of extract followed by EDE (777.77 mg ascorbic acid equivalents/g sample), EDM (770.37 mg ascorbic acid equivalents/g sample), EDA (711.11 mg ascorbic acid equivalents/g sample) and EDH (688.88 mg ascorbic acid equivalents/g sample) as shown in Fig. 3. There was recorded a significant correlation between the reducing power and with both TPC (R^2^ = 0.9812, *P* < 0.01) and TFC (R^2^ = 0.7349, *P* < 0.05) shown in Table 6.

#### Total antioxidant capacity (phosphomolybdenum assay)

**Fig. 4:**
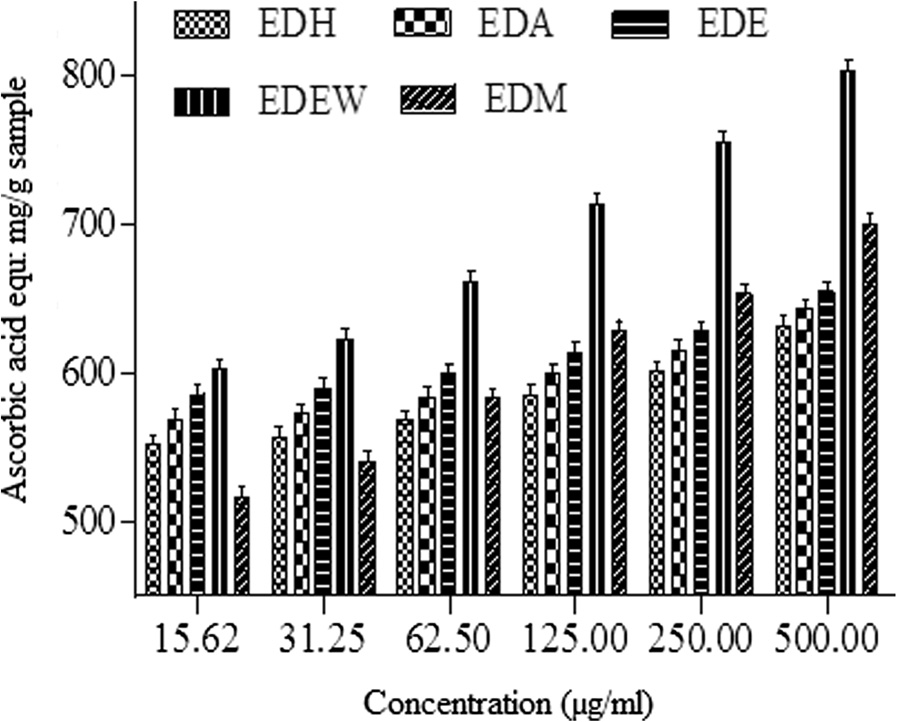
Total antioxidant activity of different concentrations of *Euphobia dracunculoids* extracts

Total antioxidant capacity of various extracts was determined by phosphomolybdate method and expressed as equivalents of ascorbic acid (mg/g of extract) as shown in Fig. 4. Total antioxidant capacity was found to decrease in the order, EDEW > EDE > EDM > EDA > EDH. All the samples exhibited an increase in antioxidant capacity with increase in concentration. The assay showed significant correlation with TPC (R^2^ = 0.7429, *P* < 0.05) and TFC (R^2^ = 0.893, *P* < 0.01) as shown in Table 6.

### Anti-inflammatory activity of *E. dracunculoides*

The results of carrageenan-induced rat paw edema are summarized in Table 7. EDH, EDA, EDE, EDEW and EDM of *E. dracunculoides* were tested for their anti-inflammatory effects. The results obtained indicated that EDH possessed significant (*P* < 0.05) anti-inflammatory activity in rats followed by EDE and EDEW. The EDH at the test dose i.e. 300 mg/kg body weight reduced the carrageenan-induced edema up to 68.660 ± 10.502 % whereas the standard drug diclofenac sodium showed 78.823 ± 6.395 % of edema inhibition after 4 h of carrageenan injection. Similarly EDE and EDEW showed 51.384 ± 8.623 % and 46.302 ± 8.975 % inhibition of edema formation after 4 h of carrageenan administration.

**Table 7:** Effect of *E. dracunculoides* on carrageenan-induced paw edema in rat

Treatment mg/kg	Edema volume (ml)/ Percent edema inhibition				
	0 h	1 h	2 h	3 h	4 h
DMSO (2 ml)	0.506 ± 0.046	0.776 ± 0.045	0.710 ± 0.021	0.671 ± 0.017	0.600 ± 0.021
DIC (10)	0.551 ± 0.056	0.706 ± 0.056	0.640 ± 0.049	0.613 ± 0.049	0.586 ± 0.056
		42.862 ± 5.614^ab^	56.903 ± 8.443^ab^	69.975 ± 5.730^a^	78.823 ± 6.395^a^
EDH (300)	0.548 ± 0.022	0.685 ± 0.033	0.630 ± 0.021	0.615 ± 0.018	0.600 ± 0.023
		49.652 ± 7.649^a^	60.171 ± 3.690^a^	67.524 ± 8.004^a^	68.660 ± 10.502^a^
EDA (300)	0.548 ± 0.035	0.751 ± 0.027	0.696 ± 0.030	0.683 ± 0.032	0.651 ± 0.031
		24.960 ± 7.283^c^	27.492 ± 5.730^d^	34.028 ± 9.170^c^	37.156 ± 6.297^c^
EDE (300)	0.560 ± 0.037	0.730 ± 0.016	0.676 ± 0.037	0.651 ± 0.039	0.640 ± 0.026
		37.307 ± 8.113^abc^	43.014 ± 12.271^bc^	55.269 ± 7.215^ab^	51.384 ± 8.623^b^
EDEW (300)	0.520 ± 0.046	0.705 ± 0.045	0.643 ± 0.030	0.633 ± 0.044	0.608 ± 0.039
		31.752 ± 3.883^bc^	39.746 ± 10.125^cd^	44.648 ± 9.127^bc^	46.302 ± 8.975^bc^
EDM (300)	0.545 ± 0.032	0.738 ± 0.026	0.683 ± 0.031	0.676 ± 0.026	0.653 ± 0.039
		28.665 ± 11.396^c^	32.394 ± 8.443^cd^	35.662 ± 12.938^c^	34.107 ± 8.975^c^

*EDH E. dracunculoides* n-hexane extract, *EDA E. dracunculoides* aqueous extract, *EDE E. dracunculoides* ethyl acetate, *EDEW E. dracunculoides* ethyl acetate + water extract, *EDM E. dracunculoides* methanol extract (EDM), *DIC* diclofenac sodium

Data values shown represent mean ± SD (*n* = 6). Means with different superscript (^a-d^) letters in the column are significantly (*P <* 0.05) different from one another

### Analgesic activity of *E. dracunculoides*

In this assay *E. dracunculoides* extracts; EDH, EDA, EDE, EDEW and EDM were evaluated in rats for their analgesic potential in hot plate test (Table 8). All the extracts showed increase in the percent latency period with the maximum was recorded with 74.309 ± 5.864 % for EDH as compared to 78.889 ± 5.853 % with morphine after 60 min of drug administration. The EDE and EDM also exhibited more than 50 % of analgesia. The extracts and the standard drug showed less analgesia after 90 min of drug administration except the EDA and EDEW where more analgesia was recorded after 90 min of drug administration.

**Table 8:** Analgesic activity of *E. dracunculoides* of hot plate test in rat

Treatment (mg/kg)	Latency time sec				Percent analgesia		
	0 h	30 min	60 min	90 min	30 min	60 min	90 min
DMSO (2 ml)	9.000 ± 0.894	9.833 ± 1.169	10.167 ± 0.752	10.167 ± 0.752	3.971 ± 3.588^d^	5.490 ± 3.378^e^	5.490 ± 3.378^e^
Morphine (10)	9.333 ± 1.032	21.000 ± 1.549	25.667 ± 1.032	22.500 ± 1.048	56.160 ± 9.114^b^	78.889 ± 5.853^a^	63.632 ± 5.449^ab^
EDH (300)	9.833 ± 1.169	21.667 ± 1.633	24.833 ± 1.169	24.000 ± 1.095	58.304 ± 9.974^a^	74.309 ± 5.864^b^	70.206 ± 5.445^a^
EDA (300)	9.666 ± 0.816	14.167 ± 1.602	15.167 ± 1.169	16.833 ± 0.752	22.164 ± 6.680^c^	26.964 ± 6.193^d^	35.223 ± 3.210^d^
EDE (300)	10.000 ± 0.894	16.000 ± 1.414	22.333 ± 1.211	21.333 ± 1.032	29.804 ± 8.470^bc^	61.811 ± 4.528^b^	56.508 ± 6.363^bc^
EDEW (300)	9.666 ± 0.816	15.500 ± 1.048	19.333 ± 1.633	19.667 ± 1.032	28.676 ± 4.528^bc^	47.331 ± 9.410^c^	49.204 ± 4.254^c^
EDM (300)	9.666 ± 0.816	17.833 ± 1.472	20.000 ± 1.788	19.500 ± 1.048	40.196 ± 6.553^b^	50.959 ± 7.486^bc^	48.248 ± 5.943^c^

*EDH E. dracunculoides* n-hexane extract, *EDA E. dracunculoides* aqueous extract, *EDE E. dracunculoides* ethyl acetate, *EDEW E. dracunculoides* ethyl acetate + water extract, *EDM E. dracunculoides* methanol extract

Data values shown represent mean ± SD (*n* = 6). Means with different superscript (^a-e^) letters in the column are significantly (*P <* 0.05) different from one another

## Discussion

The role of medicinal plants in curing diseases is increasing due to the presence of versatile compounds that have the ability to cure a variety of diseases and helping physicians to cope with increasing ratio of ailments these days [34]. Plants are rich in variety of compounds with different polarities [35]. Maximum yield was obtained in EDEW followed by EDM and the minimum yield was recorded in EDE following the rule that polar solvents dissolve more compounds in comparison to that of non-polar in short time at room temperature [36]. Present studies are in agreement to the concept of polarity based extraction with the experiment run by [37] on *Maytenus royleanus* leaves and [38] evaluating *Rumex hastatus* roots.

Preliminary qualitative phytochemical screening gives a clue for the medicinal aptitude of the herb. In the conducted study bioactive components that impart biologically active nature to the plant were screened and results ensured the presence of terpenoids, coumarins, flavonoids, tannins, phenols, alkaloids, saponins, and betacyanin. These variant compounds ranging from low polarity to high polarity were extracted in their respective solvents.

The EDH was analyzed through GC-MS and it was found to contain 30 chemical constituents eluted between 6.36 and 40.10 min. Maximum proportion was contributed by terpenoids followed by lactones, steroids, phenols and heterocycles. GC-MS analyses of EDH validated the concept of polarity based extraction as the most of the compounds identified were of non-polar nature.

In the present study through HPLC analysis of EDEW presence of four compounds; rutin, catechin, caffeic acid and quercetin was evaluated. Rutin was present in maximum amount (65.8 ± 2.2 μg/mg dry extract) followed by catechin (15.3 ± 1.2 μg/mg dry extract). Rutin is a known and well reputed secondary metabolite of plants with admirable hepatoprotective, anti-inflammatory and antioxidant activities [39]. Another well-known polyphenolic compound, catechin has good antioxidant potential and provides a reliable defense wall against free radicals. Catechin provides protection against neurological disorders, inflammation and apoptosis. The admiring amount of standard antioxidant phenolic compounds in EDEW is the justification of raised antioxidant potential of this extract. Significant correlation between TPC and TFC with that of antioxidant assays also validates the HPLC results.

Natural antioxidants have attained worth reputation in treating several diseases and have raised the value of folk herbal medicines in the modern era. The ability of DPPH radical scavenging is considered as a milestone while assessing the antioxidant aptitude of a crude plant extract or an isolated pure compound. It is a very short timed assay for appraising the antioxidant abilities and has worth economic values [40]. In this study EDEW showed the maximum scavenging activity with lowest IC_50_ value, followed by EDE < EDM < EDA < EDH. Greater quantity of phenolics and flavonoids extracted in EDEW might exhibit good scavenging abilities due to donation of electron or hydrogen to stabilize DPPH free radicals. None of the five extracts showed IC_50_ below ascorbic acid used as standard. Though [41] and [42] used different techniques for extraction yet our results are consolidating their findings. The results reported in this study indicated a good correlation with the TFC while non-significant correlation was recorded with TPC. The strong correlation of IC_50_ values with TFC might be attributed by the presence of active flavonoids such as myricetin, rutin, caffeic acid and catechin.

Antioxidant potential of a plant crude extract or compound can also be estimated by its ability to oxidize linoleic acid. β-carotene is a fat soluble hydrocarbon with yellowish orange color. Linoleic acid hydroperoxides on reaction with β-carotene bleaches its color. This is due to the fact that β-carotene forms a complex with linoleic acid and oxidizes it. This consumption of β-carotene causes the reduction of bright yellow color to light milky color. Now the presence of an antioxidant in the reaction mixture checks the consumption of β-carotene by acting on linoleic acid free radicals. As the β-carotene is released from the complex between it and linoleic acid, the yellow color of the reaction mixture is regained. Hence stronger the antioxidant present in the reaction mixture brighter will be the color and higher will be its optical density [43]. In the present study, EDEW and EDE showed the best activity with lower IC_50_ values. Significantly good correlation of IC_50_ values was observed with TPC (*P* < 0.01) and TFC (*P* < 0.05) which defines that more active constituents exhibiting inhibition of β-carotene bleaching belong to the TPC while constituents having moderate activity are present in TFC.

Hydroxyl radical is generated during various biochemical reactions in the body. It is a short lived, very toxic free radical having affinity for biomolecules like lipids, proteins, amino acids, sugars, deoxyribonucleic acids, leading to cancer, mutagenesis and cytotoxicity [44]. Superoxide dismutase converts superoxide radical into hydrogen peroxide which is converted to a highly reactive hydroxyl radical. Evidence of •OH scavenging activity by various extracts was obtained through deoxyribose system [45]. *In vivo* hydroxyl radicals are probably produced through Haber–Weiss reaction where Fe^+3^ is reduced to Fe^+2^ with the help of O_2_^•-^ which leads to initiation of Fenton reaction between H_2_O_2_ and Fe^+2^ [46]. In the present study EDEW and EDE extracts showed the best activity with lower IC_50_ values. A significant correlation of IC_50_ values was present with both TPC and TFC. Our results are in well accordance to the findings of [47] who reported methanol extract of *Dicliptera roxburghiana* as the most active to scavenge hydroxyl radicals.

At pH 7.4 a vigorous generation of nitric oxide from sodium nitroprusside occurs which further in favorable conditions viz aerobic conditions and aqueous solution react with oxygen and convert to nitrite ions, that can be appraised by Griess reagent. These nitrite ions impart pink color to the reaction mixture. Entities with NO^−^ scavenging abilities hinder the nitrite ion production by consuming the available oxygen. In our study EDEW and EDE showed comparatively good results than rest of the extracts and significant correlation of IC_50_ values was observed with TPC and TFC. This is due to the fact that EDEW and EDE are rich in bioactive phenolic and polyphenolics which have a fine tendency to scavenge NO^−^ free radicals that are responsible for oxidative stress. Research of [48] and [49] is in agreement to our findings.

Idea of iron chelating assay is based on the principle of scavenger’s ability to decolorize the iron-ferrozine complex. Ferrozine quickly reacts with the iron (II) to form water soluble colored complex. Scavenging entity present in the extract forms chelates with iron (II) hindering the iron-ferrozine complex formation and ultimately lowering the color intensity of the solution. In the present study EDE exhibited the best performance with lowest IC_50_ among all extracts in comparison to EDTA used as standard. Significant correlation of IC_50_ values was expressed with TFC (*P* < 0.01) and with TPC (*P* < 0.05) indicating that more active flavonoids constituents for iron chelation activity.

The basic principle of phosphomolybdate assay is that antioxidant species reduces Mo (IV) to Mo (V) and this reduced form of Mo forms complex with phosphate at acidic pH and raised temperature impart dark green color to the final solution [50]. The electron/ hydrogen donating pattern of antioxidants depends upon its structure and series of redox reactions occurring in the activity [51]. EDEW and EDE showed admiring results than that of EDM, EDA and EDH. The assay showed a good correlation with TPC as well as TFC. Jan et al. [52] also reported aqueous extract as the best extract in phosphomolybdenum assay, followed by methanol. Moreover, significant correlation has also been reported with TPC which is in consensus with the present study but the method of extraction was a bit different, which suggests that this methodology made no difference in extracting bioactive compounds responsible for total antioxidant activity of the plant extract.

Reducing power of *E. dracunculoides* was determined by using the potassium ferricynide reduction method. In this assay iron (Fe^+3^) in ferric chloride is converted to ferrous (Fe^+2^) by antioxidant compound/extract resulting in conversion of yellow color of the test solution to green. The intensity of green colour is directly proportional to the reducing power of the sample. Basically reducing power of the sample is due to hydrogen donating ability of antioxidants to the free radicals [53]. The assay was significantly correlated with TPC and TFC. This assay results in a pattern of EDEW > EDE > EDM > EDA > EDH at 250 μg/ml. Our study has been supported by the report of [38] that crude methanol extract was the best sample in reducing power assay after butanol.

Carrageenan is widely used to induce paw edema in rodents to demonstrate anti-inflammatory effect of drugs or herbs. Carrageenan-induced inflammation is considered to be a biphasic model. Initial stage (1–2 h) contributes to the release of histamine, bradykinin and serotonin which mediates the increased synthesis of prostaglandins from surrounding tissues of the injured site. The later phase (3–4 h) is characterized by the elevated level of prostaglandins mediated by the elevated release of leukotrienes and bradykinin. During this phase the cyclo-oxygenase-2 (COX-2) converts arachidonic acid into prostaglandins which is a key factor of inflammation maintenance. In this experiment carrageenan induced edema in the hind paw of rats was inhibited by all the extracts. EDH significantly inhibited the edema formation; 1, 2, 3 and 4 h after the injection of carrageenan in the hind paw of rats. These results indicated that the phyto-constituents present in EDH inhibited the inflammatory mediators of the initial as well as late phase of inflammation induced with carrageenan. The other extracts however, more effectively inhibited the edema during the initial phase. Steroids are established anti-inflammatory agents which inhibit the production of prostaglandin not only by inducing the biosynthesis of phospholipase A_2_ inhibitor but also by raising the level of cyclo-oxygenase/PGE isomerase [54–56]. In the present investigation GC-MS analysis of EDH revealed the existence of stigmasta-5, 22-dien-3-ol, (3á,22E)- (CAS); a phytosterol possesses strong anti-inflammatory and analgesic properties probably contributing towards the anti-inflammatory properties of EDH [57, 58]. The standard drug also exhibited the strong anti-inflammatory potential after 2, 3 and 4 h of carrageenan injection to rats. Diclofenac sodium like other NSAIDs targets the COX-2 enzyme thereby inhibiting the formation of the paw edema. The anti-inflammatory results obtained with EDH in both phases might be attributed by the counteraction of anti-inflammatory agents such as sterols and terpenoids [59]. The anti-inflammatory effects obtained by the polar extracts; EDE, EDA, EDEW and EDM during the initial phase might be attributed by the presence of flavonoids (rutin, catechin, caffeic acid and myricetin) and other constituents. Anti-inflammatory activities of rutin, catechin and caffeic acid have been well documented [60]. Our studies are in consensus with [60] and [61, 62] who generated the same anti-inflammatory results of *Acacia hydaspica* and *Boerhavia procumbens* in rats.

Thermal nociception models such as, hot plate test was used to evaluate the central analgesic activity. In this study all the extracts of *E. dracunculoides* showed analgesic effect in the hot plate test. EDH exhibited the best potent analgesic activity among the extracts with 74.309 ± 5.864 % inhibition of pain sensation followed by EDE (61.811 ± 4.528 %) in comparison to morphine (78.889 ± 5.853 %) after 60 min of drug administration. Morphine induces analgesic effect through activation of opioid receptors and the apparent similarity between the results of extracts with standard morphine, indicates that they might work in a same manner to reduce pain sensation. The presence of steroids in EDH might induce the biosynthesis of phospholipase A_2_ inhibitor and cyclo-oxygenase/PGE isomerase which in turn inhibits the pain producing prostaglandins. Our results are in agreements to the findings of Mondal et al. [63] evaluating *Alternanthera sessilis* as analgesic agent. Backhouse et al. [64] also reported the same results while evaluating *Buddleja globosa* as analgesic and anti-inflammatory agent.

## Conclusion

The present results suggest that the flavonoids in *E. dracunculoides* might be the key players in scavenging of oxidative stress inducing species while flavonoids along with sterols and terpenoids alleviate the inflammation and pain inducing mediators.
